# Microwave-Sintered Lunar Regolith Bricks for Lunar Infrastructure: Fracture Behavior, Tribological Performance, and Electromagnetic Wave Transmission

**DOI:** 10.3390/ma19091907

**Published:** 2026-05-06

**Authors:** Kelei Zhu, Juntao Guo, Qiqi Ning, Zhaobo Han, Longxiang Xu, Zhen Liu, Bo Gao, Jinping Li

**Affiliations:** 1 School of Astronautics, Harbin Institute of Technology, Harbin 150080, China; 2023111050@stu.hit.edu.cn (K.Z.); guojuntao2002@163.com (J.G.); 2023111349@stu.hit.edu.cn (Q.N.); 24s118104@stu.hit.edu.cn (Z.H.); luoxue2741216369@outlook.com (L.X.); unliuzhen@163.com (Z.L.); 20230194@hit.edu.cn (B.G.); 2 National Key Laboratory of Science and Technology on Advanced Composites in Special Environments, Harbin Institute of Technology, Harbin 150080, China

**Keywords:** microwave sintering, lunar regolith bricks, fracture behavior, tribological performance, electromagnetic wave transmission

## Abstract

Microwave-sintered lunar regolith bricks are promising candidates for in situ construction of lunar infrastructure, where structural load-bearing capacity and multifunctional performance are simultaneously required. Currently, there remains a research gap concerning the service performance of microwave-sintered lunar soil bricks under predictable load-bearing, wave-transparent, and friction working conditions. In this study, lunar bricks were fabricated at different microwave sintering temperatures, and the effects of temperature on their microstructure and engineering properties were systematically investigated. The sample sintered at 1000 °C achieved a density of 2.96 g/cm^3^ and a compressive strength of 260 MPa. Combined experimental observations and numerical simulations revealed a typical brittle fracture behavior, primarily governed by residual porosity within the material. Tribological tests showed a low wear rate of 6.51 × 10^−5^ mm^3^/(N·m), indicating good wear resistance and potential applicability for lunar road paving. Dielectric measurements in the X-band (8.2–12.4 GHz) demonstrated a high electromagnetic wave transmittance ranging from 49.8% to 94.6%, suggesting suitability for communication-related or protective wall structures. These results demonstrate that microwave sintering effectively enhances the densification of lunar regolith while enabling the coordinated optimization of mechanical, tribological, and electromagnetic properties, providing practical guidance for the design of multifunctional materials for lunar infrastructure construction.

## 1. Introduction

The establishment of a permanent lunar base represents a critical milestone toward sustained human presence and long-term scientific exploration on the Moon [1,2,3]. Key infrastructure components of a lunar base include roads, landing platforms, protective walls, and habitable shelters. To achieve sustainable and economically viable construction, extensive utilization of in situ lunar regolith for building materials is essential, thereby significantly reducing the dependence on material transportation from Earth [4]. In recent years, microwave sintering has attracted considerable attention for the fabrication of lunar regolith-based bricks [5,6]. Compared with conventional sintering methods, microwave sintering offers advantages such as lower energy consumption, shorter processing time, simplified processing routes, and compatibility with additive manufacturing technologies [7,8]. Previous studies have demonstrated a strong coupling between microwave energy and lunar regolith, enabling rapid heating and densification within a short duration [7]. Moreover, when microwave sintering is applied to lunar regolith simulants, the introduction of SiC susceptors facilitates a synergistic heating mechanism combining surface radiation and volumetric heating, resulting in relatively uniform, dense, and structurally intact lunar brick materials [9,10,11,12,13,14,15,16,17]. However, existing studies have primarily focused on optimizing processing parameters, such as microwave power, sintering temperature, and simulant type, while systematic investigations into fracture behavior and failure mechanisms remain limited [5,18].

From an engineering application perspective, a comprehensive understanding of the failure behavior of lunar bricks is crucial for assessing their structural stability and service reliability [19]. In particular, for load-bearing components such as protective walls and shelters, fracture behavior directly governs structural safety under mechanical loading conditions [20]. In addition to structural applications, lunar bricks are also expected to serve as paving materials for roads and landing platforms, which constitute fundamental infrastructure elements during the early stages of lunar base construction. Under such service conditions, tribological performance, including wear resistance and durability, becomes a key factor influencing long-term operational reliability [21]. Furthermore, for shelter structures, electromagnetic wave transmission characteristics must be carefully considered, as they directly affect communication signal propagation between internal and external environments, as well as Earth–Moon communication links. Insufficient wave transmission performance may lead to signal attenuation or communication interruption, posing risks to mission safety and operational efficiency [22]. Therefore, from an engineering standpoint, lunar brick materials should be evaluated through a coordinated optimization of mechanical, tribological, and electromagnetic performance.

To address these challenges, lunar regolith bricks were fabricated at different microwave sintering temperatures in this study. The effects of sintering temperature on microstructure, mechanical properties, fracture behavior, X-band electromagnetic wave transmission, and friction and wear performance were systematically investigated. This work aims to provide a comprehensive understanding of the service behavior of microwave-sintered lunar bricks from a multifunctional perspective, offering both material-level insights and engineering design references for future lunar infrastructure construction.

## 2. Experimental Section

### 2.1. Material and Preparation

The mare-type lunar regolith simulant (CLRS-1A) used in this study was supplied by the Guiyang Institute of Geochemistry, Chinese Academy of Sciences. The simulant is classified as low-titanium lunar regolith and was produced from basaltic volcanic rock through impurity removal, crushing, and grinding processes. The median particle size of the simulant was approximately 82 μm, and its primary mineral phases consisted of volcanic glass, olivine, pyroxene, and plagioclase.

For sample preparation, 200 g of lunar regolith simulant was mixed with 20 g of distilled water and homogenized in a mortar for 5 min to ensure uniform wetting. The distilled water served solely as a temporary binder to provide sufficient green strength for compaction and did not participate in the subsequent sintering process. After mixing, the slurry was placed into a mold and uniaxially pressed at 5 MPa for 5 min to form green bodies. The green bodies were first air-dried for one week, followed by oven drying at 120 °C for 12 h to completely remove residual moisture prior to sintering. After drying, the green bodies were sintered in a vacuum microwave sintering furnace. The microwave power was maintained at 660 W throughout the process. A stepwise heating strategy was adopted, in which the temperature was held for 3 min at every 50 °C increment after reaching 750 °C, until final sintering temperatures of 900 °C, 950 °C, and 1000 °C were achieved. The corresponding samples were denoted as L-900, L-950, and L-1000, respectively.

### 2.2. Characterization

The internal pore structure of the sample was characterized using X-ray computed tomography (X-CT, Nanovoxel 1000, Tianjin Sanying Precision Instrument Co., Ltd., Tianjin, China) with cubic specimens measuring 1.5 × 1.5 × 1.5 mm^3^. After scanning, the projection images were reconstructed into serial grayscale slices using the instrument software, followed by three-dimensional rendering and segmentation of the pore phase from the solid matrix. The reconstructed datasets were then used to analyze pore-related descriptors such as total porosity, pore size distribution, pore morphology, and pore-network connectivity.

Quasi-static compressive properties (DF13.204D, Sinotest Equipment Co., Ltd., Changchun, China) were measured using a universal testing machine at a constant displacement rate of 0.5 mm/min. Cylindrical specimens with a diameter of 5 mm and a height of 10 mm were employed. During testing, the applied load and crosshead displacement were continuously recorded, and the compressive response was evaluated from the stress–strain behavior, where engineering stress was calculated from the applied load divided by the initial cross-sectional area. The compressive strength was taken from the maximum stress reached during loading. To capture deformation heterogeneity during compression, digital image correlation (DIC) was applied within the selected area of interest (AOI). Before testing, a high-contrast random speckle pattern was prepared on the specimen surface, and sequential images acquired during loading were correlated with the undeformed reference image to obtain full-field displacement and strain maps. The microstructural features of fractured and surfaces were examined using scanning electron microscopy (SEM, SU5000, Hitachi, Tokyo, Japan).

Dielectric properties in the X-band frequency range were measured using a vector network analyzer (PNA-N5234A, Agilent, Santa Clara, CA, USA) with rectangular specimens of 22.9 × 10.2 × 2 mm^3^, as this frequency band is widely used in space communication, radar systems, and structural enclosures in aerospace applications, making it particularly relevant for evaluating the electromagnetic compatibility of lunar infrastructure materials.

Tribological performance was evaluated using a multifunctional tribometer (MFT-5000, Rtec Instruments, Geneva, Switzerland) under a sliding speed of 0.1 m/s, a normal load of 10 N, and a total sliding distance of 60 m. A tungsten carbide (WC) ball with a diameter of 6.35 mm was used as the counterface. The friction test protocol was refined with reference to ASTM G133. During testing, the friction force was continuously recorded and converted into the coefficient of friction as a function of sliding distance or time. After testing, the wear scar and wear track were characterized by microscopy or profilometry to evaluate wear morphology, wear volume, and specific wear rate. In the context of lunar road and landing platform materials, this test provides a simplified laboratory approximation of repeated mechanical contact and abrasive service conditions.

## 3. Results and Discussion

### 3.1. Fracture Mechanism

Table 1 summarizes the density, hardness, and compressive strength of the samples sintered at different temperatures. With increasing sintering temperature, all samples exhibit pronounced improvements in densification and mechanical performance. When the sintering temperature increased from 900 °C to 950 °C, the density rose from 2.85 to 2.93 g/cm^3^, accompanied by a substantial increase in hardness from 7.85 to 13.26 GPa. More notably, the compressive strength increased sharply from 51.25 MPa to 196.01 MPa, reflecting an accelerated densification process driven by enhanced mass transport and interparticle bonding. When the sintering temperature was further increased to 1000 °C, the density increased only slightly to 2.96 g/cm^3^, whereas the hardness and compressive strength continued to rise, reaching 14.11 GPa and 260.36 MPa, respectively. This behavior indicates that the material approached a near-fully densified state, in which further temperature elevation primarily strengthens intergranular bonding and load-bearing capacity rather than producing significant additional bulk densification [23].

**Figure 1:**
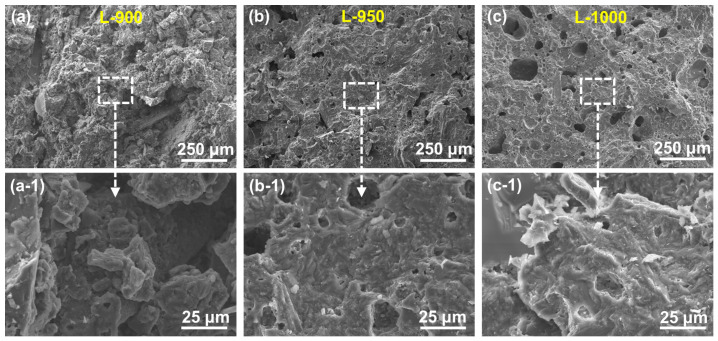
SEM images of the cross sections for samples sintered at different temperatures: (**a**,**a-1**) 900 °C, (**b**,**b-1**) 950 °C, and (**c**,**c-1**) 1000 °C.

Figure 1 presents cross-sectional SEM images of samples sintered at different temperatures. The sample sintered at 900 °C exhibits a loose particulate microstructure, indicating that the sintering driving force at this temperature is insufficient to achieve effective densification. When the sintering temperature is increased to 950 °C, the microstructure becomes significantly more compact, with enhanced particle bonding and reduced pore connectivity, reflecting intensified mass transport and microstructural rearrangement during sintering. At a sintering temperature of 1000 °C, the overall microstructure appears highly consolidated; however, isolated circular pores are observed in localized regions. These pores are likely associated with localized melting or gas release at elevated temperatures, followed by pore entrapment upon cooling [24]. Although the presence of such pores introduces microscale heterogeneity in local density, they do not markedly degrade the macroscopic mechanical performance of the material. On the contrary, the formation of transient liquid phases at high temperatures enhances intergranular bonding and structural integrity, contributing to the observed high strength. This behavior suggests that elevated sintering temperatures facilitate liquid-phase-assisted mass transport, which promotes grain boundary migration and bonding, thereby improving the overall load-bearing capacity of the lunar regolith bricks [25].

**Figure 2:**
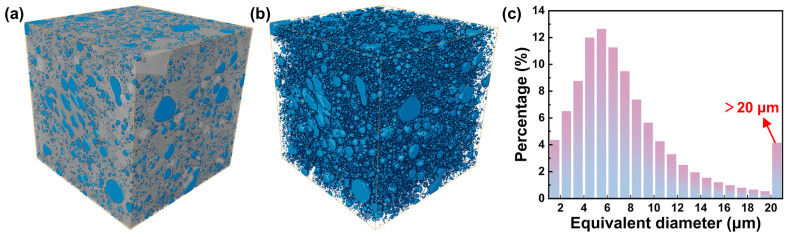
(**a**,**b**) Pores distribution of L-1000 sample. (**c**) The proportion of pores of different diameters.

To further clarify the origin of the high compressive strength and fracture characteristics of the L-1000 sample, three-dimensional reconstruction of its internal pore structure was performed using X-ray computed tomography, as shown in Figure 2. As illustrated in Figure 2a,b, the pores within the L-1000 sample are predominantly isolated or semi-connected, with a largely spherical morphology, consistent with the SEM observations. The total porosity of the sample is approximately 16.77%, indicating that high-temperature microwave sintering effectively promotes densification while retaining a limited amount of residual porosity. Importantly, the relatively low connectivity and dispersed spatial distribution of pores impede crack coalescence and long-range crack propagation under compressive loading. As a result, the material maintains excellent macroscopic compressive strength despite the presence of residual pores. The pore size distribution shown in Figure 2c further reveals that the equivalent diameters of most pores are concentrated in the range of 0–12 μm, with only a small fraction exceeding 20 μm. Such pores are expected to act as local stress concentrators during mechanical loading, potentially serving as initiation sites for microcrack formation [26]. However, due to their limited size and spatial isolation, these microcracks are unlikely to rapidly propagate or merge, which is consistent with the observed high strength and subsequent brittle failure behavior.

**Figure 3:**
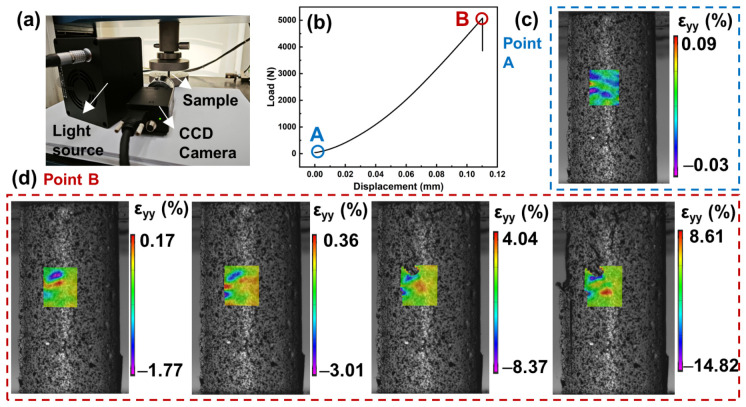
(**a**) Compressive strength testing system with DIC technology. (**b**) Load–displacement curve of L-1000 sample. (**c**,**d**) The strain contour map in the y-direction of AOI.

Following the characterization of pore distribution in the L-1000 sample, digital image correlation (DIC) was employed to monitor the evolution of local strain fields during uniaxial compression, as shown in Figure 3, in order to elucidate its deformation response and fracture behavior under load. A schematic of the DIC testing setup is presented in Figure 3a, in which a CCD camera was used to capture surface deformation images in real time and calculate the corresponding strain fields. The load–displacement curve in Figure 3b indicates that the sample initially exhibits a linear elastic response. With increasing displacement, the applied load rises steadily and reaches a maximum at point B, which corresponds to the onset of macroscopic instability and failure. The associated strain contour maps reveal that at the early loading stage (point A), the strain distribution on the sample surface is relatively uniform, with only minor localized strain concentrations. As loading progresses toward point B, a distinct circular compressive strain localization zone emerges, as shown in Figure 3d. These localized strain zones are typically located near pores or microstructural defects, indicating pronounced local stress concentration effects [17]. With further loading, the compressive strain localization zone expands and intensifies, eventually defining the dominant crack initiation and propagation path. The accumulation and coalescence of localized deformation within this high-strain region ultimately led to macroscopic fracture of the sample, consistent with a typical brittle failure process governed by microstructural heterogeneity. More detailed quantitative treatment of strain-evolution metrics or fracture-energy-related parameters is beyond the scope of the current study.

**Figure 4:**
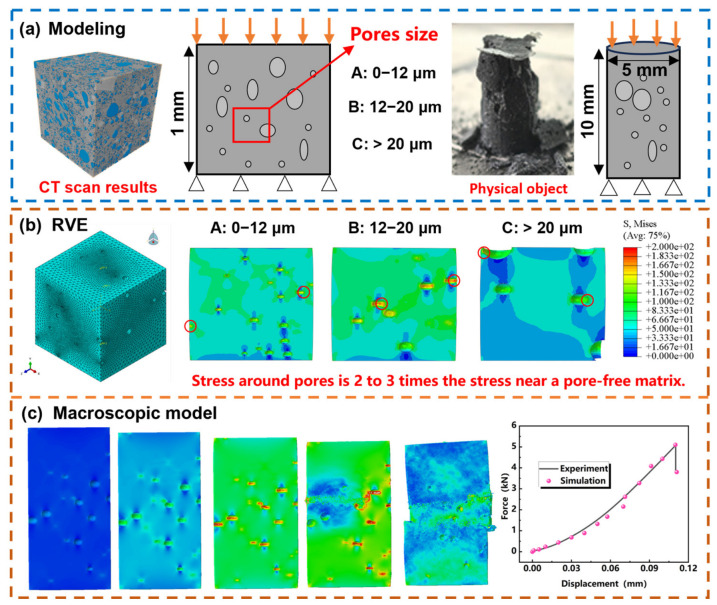
Finite element analysis results: (**a**) modeling strategy, (**b**) stress distribution around pores of different sizes, and (**c**) stress-localization evolution in the specimen under compression.

As shown in Figure 4a, representative volume element (RVE) models and an actual specimen model have been constructed to characterize the microscopic pore structure of the material. The calculations were performed using an explicit dynamic step. 

The RVE model was established based on the CT scan results shown in Figure 3a. The matrix is a cube with dimensions of 1 mm × 1 mm × 1 mm, within which circular pores of three size ranges are randomly generated. During the modeling process, by controlling the total volume of randomly generated pores and the inter-pore spacing, the total porosity of each model was made to be largely consistent with the experimentally measured values. For boundary conditions, a z-direction displacement constraint was applied only to the bottom surface of the matrix. The loading method adopted z-direction force loading, applied to the top surface of the model. The model was meshed using improved quadratic tetrahedral elements of type C3D10M. Models A, B, and C consist of 833,232, 151,529, and 67,576 elements, respectively. The material parameters of the matrix were obtained from experimental test results, with a Young’s modulus of 19.15 GPa and a Poisson’s ratio of 0.23. The material failure criterion adopts the maximum principal stress criterion, and the failure stress is taken as the experimentally measured value of 265 MPa. The geometric dimensions of the specimen are consistent with those used in the compression tests. A z-direction displacement constraint was applied to the bottom surface of the model, and the loading method was the same as that used for the RVE model. The model was also meshed using improved quadratic tetrahedral elements of type C3D10M, comprising a total of 476,207 elements and 652,298 nodes. The material parameters were still obtained from experimental measurements, with a Young’s modulus of 19.15 GPa, a Poisson’s ratio of 0.23, and a failure stress of 254 MPa.

As shown in Figure 4b, pronounced stress concentration occurs in the vicinity of pores regardless of pore size. The stress level at the pore edges is significantly higher than that in the surrounding dense regions, and the high-stress zones tend to align along the pore connection direction, forming chain-like distributions. This behavior highlights the dominant role of pore-induced heterogeneity in governing the local stress field. These results indicate that pores act as preferential sites for stress concentration and likely failure initiation, highlighting the dominant role of pore-induced heterogeneity in governing the local mechanical response of the material.

The physical compression model is shown in Figure 4c. Based on the pore size distribution obtained from CT scanning, a pore model with pore sizes ranging from 0 to 80 μm, consistent with the scanned distribution, was established within the specimen. Displacement-force data under compressive loading were collected, and the simulated response shows reasonable agreement with the experimentally observed global compression behavior, and the predicted stress-localization pattern is consistent with the strain-localization tendency revealed by DIC. These comparisons support the use of the model for mechanism interpretation. As the applied load increases, localized stress concentration zones progressively intensify and extend between neighboring pores. The physical compression model is shown in Figure 4c. Based on the pore size distribution obtained from CT scanning, a pore model with pore sizes ranging from 0 to 80 μm, consistent with the scanned distribution, was established within the specimen. The simulated load–displacement response shows reasonable agreement with the experimental trend. As the applied load increases, localized high-stress regions progressively intensify and extend between neighboring pores. These stress-localization features indicate likely sites for failure initiation and reveal the dominant role of pore-induced heterogeneity in governing the brittle failure tendency of the specimen.

The pronounced improvement in mechanical performance with increasing sintering temperature can be interpreted in terms of temperature-activated liquid-phase-assisted densification rather than a purely phenomenological trend. Previous characterization of microwave-sintered lunar regolith simulants has shown that crystallization initiates at approximately 807 °C, while more substantial phase reorganization occurs in the 900–1100 °C range. In this temperature window, fine particles begin to soften and form initial sintering necks, and the progressively generated glassy/liquid phase fills interparticle voids and enhances grain-boundary bonding [14]. Consequently, the sharp increase in strength from L-900 to L-950 can be attributed to the transition from insufficient particle bonding to effective liquid-phase-assisted intergranular consolidation. By contrast, the relatively small density increase from L-950 to L-1000, despite the continued increase in hardness and compressive strength, suggests that the material had approached a near-fully densified state, in which further temperature elevation mainly strengthened intergranular bonding and load-bearing continuity rather than producing substantial additional bulk densification. Meanwhile, the residual pores observed in the L-1000 sample were predominantly isolated or semi-connected, which could act as local stress concentrators but were less likely to rapidly coalesce into long-range fracture paths under compression.

### 3.2. Wave Transmission Performance

**Figure 5:**
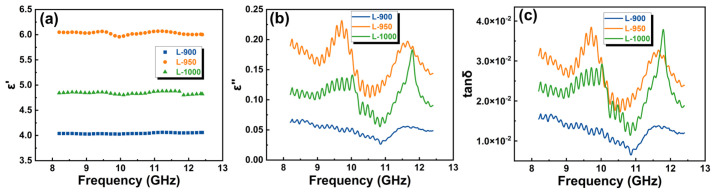
Dielectric properties of samples prepared at different sintering temperatures with a frequency of 8.2–12.4 GHz: (**a**) *ε′*, (**b**) *ε″*, and (**c**) *tan δ*.

Figure 5 illustrates the dielectric properties of samples sintered at different temperatures in the X-band frequency range of 8.2–12.4 GHz. For all samples, the real part of the permittivity (*ε′*) remains relatively stable across the investigated frequency range, indicating good electromagnetic stability within the operational band. However, the magnitude of *ε′* shows a clear dependence on sintering temperature. As shown in Figure 5a, *ε′* of the L-900 sample remains in the range of 4.0–4.1, increases markedly to approximately 5.9–6.1 for the L-950 sample, and decreases slightly to 4.8–5.0 for the L-1000 sample, while still remaining higher than that of L-900. The variation in *ε′* can be attributed to combined changes in material densification, microstructural continuity, and residual porosity induced by different sintering temperatures [27]. Moderate temperature elevation enhances polarization capability through improved interparticle bonding and microstructural compactness, leading to higher *ε′* values. At higher sintering temperatures, the formation of isolated pores associated with localized melting or gas release partially offsets this effect, resulting in a reduction in *ε′* for the L-1000 sample.

The imaginary part of the permittivity (*ε″*) and the dielectric loss tangent (*tan δ*) exhibit similar temperature-dependent trends, as shown in Figure 5b,c. The L-900 sample displays consistently low *ε″* values below 0.07 and a *tan δ* of approximately 1.0 × 10^−2^, indicating minimal dielectric loss. In contrast, the L-950 sample shows a pronounced increase in dielectric loss, with *ε″* reaching a maximum of 0.23 in the 9–11 GHz range and *tan δ* exceeding 3.7 × 10^−2^. For the L-1000 sample, both *ε″* and *tan δ* decrease to approximately 0.15 and 2.9 × 10^−2^, respectively, reflecting a reduction in dielectric loss compared with the L-950 sample.

Based on the electromagnetic wave transmission theory of a single-layer parallel dielectric slab, the relationship between transmittance (${\left|T\right|}^{2}$), frequency, and sample thickness (*d*) can be calculated using Equations (1)–(4) [28]:

(1) $$n=\frac{\varepsilon_{r}\cos\;\theta }{{\left(\varepsilon_{r}-\sin^{2}\;\theta \right)}^{0.5}}$$

(2) $$R=\frac{1-n}{1+n}$$

(3) $$\varphi =\frac{2\pi \;d}{\lambda }{\left(\varepsilon_{r}-\sin^{2}\;\theta \right)}^{0.5}$$

(4) $${\left|T\right|}^{2}=\frac{{\left(1-R^{2}\right)}^{2}\;}{{\left(1-R^{2}\right)}^{2}+4R^{2}\;\sin^{2}\;\varphi }$$

where *ε_r_* = *ε′* − *jε″*, *n* is the electromagnetic wave refraction, *R* is the reflection coefficient, *λ* is the wavelength, and *θ* is the incidence angle of the electromagnetic wave. In the calculation, it is assumed that the electromagnetic wave strikes the sample surface perpendicularly, i.e., *θ* = 0.

The transmission model adopted here is a first-order analytical approximation based on a single-layer parallel dielectric slab. This assumption was chosen to match the actual geometry of the planar rectangular specimens used in the X-band dielectric measurements, thereby maintaining consistency between the experimental configuration and the theoretical analysis. The assumption of normal incidence (*θ* = 0) corresponds to the controlled transmission-test setup and allows the dominant effects of specimen thickness and effective dielectric parameters to be isolated without introducing additional complexity from oblique incidence or geometric irregularity. In addition, the material was treated as an effective homogeneous dielectric medium, with the measured *ε′* and *ε″* representing the bulk electromagnetic response of the sintered sample in the X-band. Therefore, the present model is intended for engineering-level interpretation of thickness-dependent transmission trends, rather than for microstructure-resolved full-wave simulation. The validity of this simplified treatment is supported by the good agreement between the theoretically predicted quarter-wavelength transmittance minimum and the experimental transmittance valley observed near 3.7 mm at 10 GHz.

**Figure 6:**
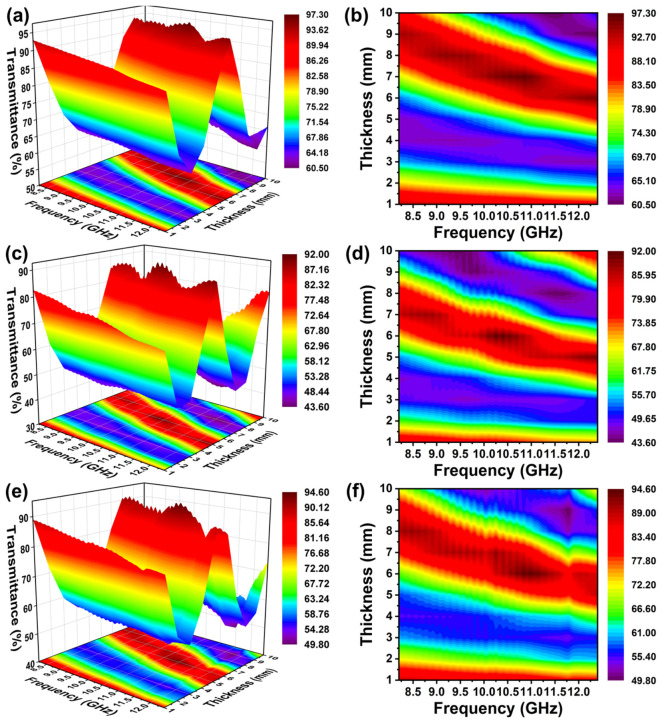
Wave transmittance diagrams with frequency (8.2–12.4 GHz) and thickness (1–10 mm) of samples prepared at different sintering temperatures: (**a**,**b**) 900 °C, (**c**,**d**) 950 °C, (**e**,**f**) 1000 °C.

Figure 6 illustrates the transmittance variation in samples sintered at different temperatures as a function of frequency and specimen thickness. As the thickness increases from 1 to 10 mm, the electromagnetic wave transmittance exhibits a pronounced periodic “high–low–high–low” oscillatory pattern, whereas only relatively mild fluctuations are observed along the frequency direction. This periodic behavior arises from multiple internal reflections within the planar dielectric medium, commonly referred to as the Fabry–Pérot resonance effect [29]. At small thicknesses (<2 mm), electromagnetic waves predominantly transmit through the samples, and multiple-reflection interference is negligible, resulting in high transmittance levels exceeding 80%. With further increases in thickness to the range of 2–6 mm, interference effects become increasingly significant, leading to a pronounced reduction in transmittance, which can decrease to approximately 43%. This behavior is consistent with the quarter-wavelength interference condition predicted by Equation (5) [28]:

(5) $$\frac{n\lambda }{4}=\frac{nc}{4\;f_{m}{\left(\varepsilon_{r}\mu_{r}\right)}^{0.5}}$$

where *n* = 1, 3, 5, …, *f_m_* is the frequency of the electromagnetic wave, *μ_r_* is the magnetic permeability of the magnetic field, which is 1, and *c* is the speed of light. When the sample thickness approaches (2n + 1)λ/4, destructive interference occurs between the transmitted and internally reflected waves, resulting in energy attenuation and a reduction in transmission efficiency. Taking 10 GHz as a representative frequency, the calculated quarter-wavelength thickness (0.25λ) is approximately 3.7 mm. The experimental results in Figure 6 exhibit a pronounced transmittance minimum near this thickness, demonstrating good agreement between theoretical predictions and experimental observations.

The L-900 sample maintains a relatively high transmittance over the entire investigated thickness range, with a minimum value of approximately 60%. In contrast, the L-950 sample exhibits pronounced transmittance minima in the thickness range of 2–6 mm, accompanied by stronger frequency-dependent fluctuations in the 9–11 GHz band. These features are consistent with the elevated dielectric loss (*ε″*) and loss tangent (*tan δ*) observed for this sample. The transmittance behavior of the L-1000 sample lies between those of L-900 and L-950, maintaining transmission levels above approximately 49% over most thicknesses from 1 to 10 mm. From an engineering application perspective, lunar regolith bricks intended for communication-related or protective wall structures must simultaneously satisfy mechanical load-bearing requirements and adequate electromagnetic wave transmission performance. Considering the combined mechanical strength and wave transmittance characteristics, a sintering temperature of 1000 °C provides the most balanced performance and is therefore identified as the optimal processing condition in this study.

### 3.3. Tribological Performance

**Figure 7:**
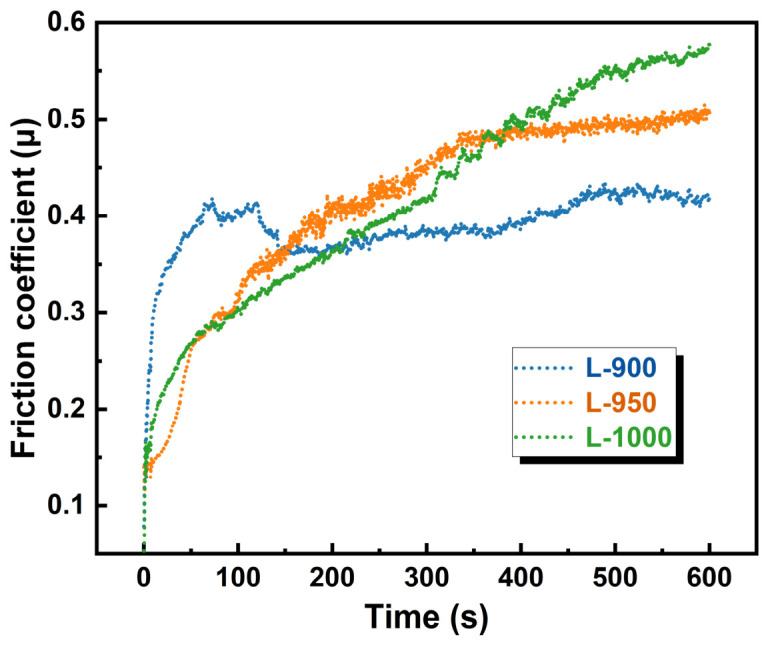
Friction coefficient of samples prepared at different sintering temperatures.

Figure 7 presents the time-dependent evolution of the friction coefficient for samples sintered at different temperatures. The L-900 sample exhibits a rapid increase in friction coefficient during the initial sliding stage, followed by pronounced fluctuations between 100 and 200 s. Subsequently, the friction coefficient stabilizes in the range of 0.35–0.40. This behavior indicates that insufficient sintering at low temperature results in relatively weak interparticle bonding, leading to repeated fracture and detachment of surface asperities during sliding. Such unstable surface degradation hinders the formation of a continuous and stable tribological layer at the contact interface. For the L-950 sample, the friction coefficient increases gradually and stabilizes at approximately 0.48, with significantly reduced fluctuations. The improved densification and interfacial bonding achieved at this sintering temperature promote the development of a more stable third-body layer, enabling more uniform load transfer and contact conditions at the sliding interface [30]. In contrast, the friction coefficient of the L-1000 sample increases continuously throughout the test, reaching approximately 0.57 at 600 s. This behavior suggests that the highly consolidated microstructure leads to increased surface brittleness under sliding conditions. During friction, the generation and accumulation of hard wear debris at the contact interface progressively increase interfacial resistance, resulting in a continuous rise in the friction coefficient [31].

**Figure 8:**
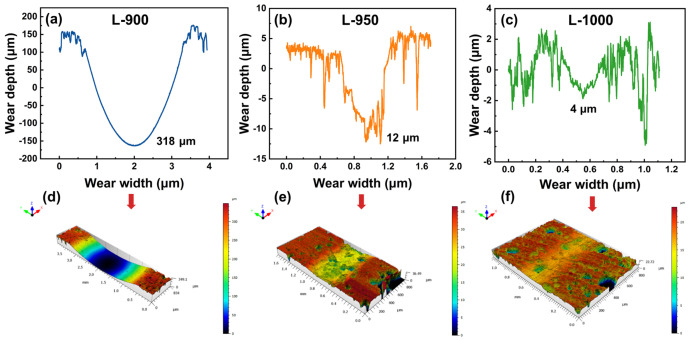
Wear scar depth and three-dimensional morphology of wear scars of samples prepared at different sintering temperatures: (**a**) 900 °C, (**b**) 950 °C, and (**c**) 1000 °C; Volumetric wear degree: (**d**) 900 °C, (**e**) 950 °C, and (**f**) 1000 °C.

The wear scar depth and three-dimensional morphologies shown in Figure 8 further corroborate the frictional behaviors discussed above. The L-900 sample exhibits the deepest wear scar, reaching approximately 318 μm, and is characterized by pronounced grooves and extensive surface spalling, indicative of severe volumetric wear. This behavior is consistent with insufficient sintering and weak interparticle bonding, which promotes material removal under sliding conditions. In contrast, the L-950 sample shows a significantly reduced wear scar depth of approximately 12 μm and a relatively uniform worn surface dominated by shallow scratches. This observation indicates that moderate sintering effectively transforms the wear mechanism from severe spalling to a more stable abrasive wear regime. The L-1000 sample exhibits the shallowest wear scar, with a depth of only about 4 μm, accompanied by fine scratches and minor surface disturbances. This behavior demonstrates that the high density and hardness achieved at this sintering temperature effectively suppress volumetric wear and enhance wear resistance.

To quantitatively evaluate the wear behavior of the samples, the wear rate (*W*) was calculated according to Equation (6) [32]:

(6) $$W=\frac{V}{FL}$$

where *V* is the wear volume, *F* is the normal load, *L* is the sliding distance. Table 2 summarizes the calculated wear rates of samples sintered at different temperatures. The L-900 sample exhibits the highest wear rate, reaching 6.04 × 10^−2^ mm^3^/(N·m), which is more than two orders of magnitude higher than those of the L-950 and L-1000 samples. In comparison, the wear rate of the L-950 sample is reduced to 5.16 × 10^−4^ mm^3^/(N·m). The L-1000 sample demonstrates the lowest wear rate, with a value of only 6.51 × 10^−5^ mm^3^/(N·m), indicating a substantial enhancement in wear resistance with increasing sintering temperature.

**Figure 9:**
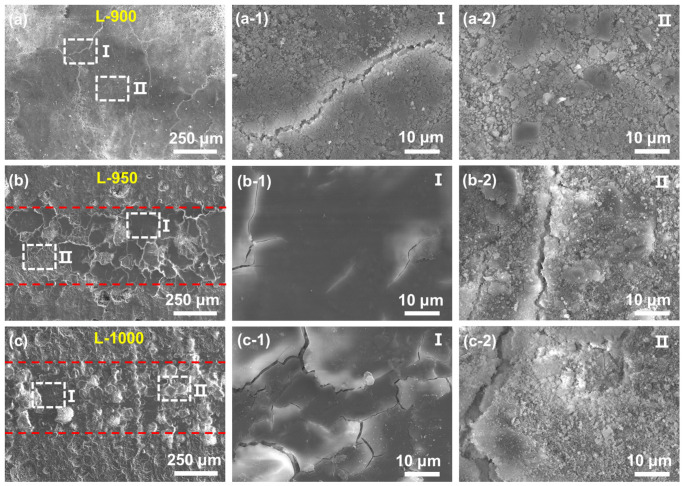
SEM images of wear scars on different samples after friction: (**a**,**a-1**,**a-2**) 900 °C, (**b**,**b-1**,**b-2**) 950 °C, and (**c**,**c-1**,**c-2**) 1000 °C.

The SEM images of the wear scars in Figure 9 reveal distinct wear mechanisms for samples sintered at different temperatures. The surface of the L-900 sample is dominated by extensive cracking and loose debris, with pronounced spalling and interfacial cracking, indicating severe material removal during sliding. This wear behavior is consistent with weak interparticle bonding and insufficient structural integrity. In contrast, the worn surface of the L-950 sample appears relatively smooth and is characterized by a fish-scale-like morphology, accompanied by only minor cracks and debris. This feature suggests the formation of a stable third-body layer at the sliding interface, which effectively mitigates material removal and contributes to improved frictional stability. The L-1000 sample exhibits a dense worn surface with localized brittle cracks and shallow spalling pits, reflecting a significantly enhanced resistance to volumetric wear. However, the repeated generation and accumulation of brittle wear debris at the contact interface led to continuous disruption and reformation of the tribological layer, which explains the relatively higher friction coefficient observed for this sample.

From an application perspective, when microwave-sintered lunar regolith bricks are considered for road paving, their low wear rate combined with a dense microstructure is expected to improve surface durability and stability, thereby supporting reliable and safe operation under long-term mission conditions.

## 4. Conclusions

This study demonstrates the feasibility of fabricating lunar regolith bricks by microwave sintering and evaluates their mechanical, tribological, and electromagnetic wave transmission performance for potential lunar infrastructure applications. The results indicate that:

1. Microwave sintering at 900–1000 °C effectively enhanced the densification and mechanical properties of the lunar regolith simulant specimens, with compressive strength increasing from 51.25 MPa at 900 °C to 260.36 MPa at 1000 °C.
2. Higher sintering temperature led to a denser microstructure with improved particle bonding, which contributed to better structural integrity and reduced wear.
3. The wear resistance improved markedly with temperature, and the L-1000 sample exhibited the lowest wear rate of 6.51 × 10^−5^ mm^3^/(N·m).
4. High X-band transmittance of 49.8–94.6% indicates that the sintered specimens can satisfy both structural and electromagnetic functional requirements.
5. Overall, the 1000 °C condition provided the best balance among mechanical, tribological, and electromagnetic properties.

It should be noted that the present work represents an initial exploration of the tribological and electromagnetic performance of microwave-sintered lunar bricks under laboratory conditions. While differences may exist between these conditions and the actual lunar environment, the results provide essential baseline data and a clear performance framework for preliminary engineering design and material selection.

## Figures and Tables

**Table 1:** Density, hardness, and compressive strength of samples sintered at different temperatures.

	L-900	L-950	L-1000
Denstity (g/cm^3^)	2.85 ± 0.0014	2.93 ± 0.0017	2.96 ± 0.0021
Hardness (GPa)	7.85 ± 0.58	13.26 ± 1.09	14.11 ± 1.13
Compressive strength (MPa)	51.25 ± 4.11	196.01 ± 12.33	260.36 ± 17.94

**Table 2:** Wear rate of samples prepared at different sintering temperatures.

	L-900	L-950	L-1000
Wear rate (mm^3^/(N·m))	6.04 × 10^−2^	5.16 × 10^−4^	6.51 × 10^−5^

## Data Availability

The original contributions presented in this study are included in the article/Supplementary Material. Further inquiries can be directed to the corresponding author.

## Supplementary Materials

The following supporting information can be downloaded at: https://www.mdpi.com/article/10.3390/ma19091907/s1, Figure S1: Temperature comparison between experiment at 3000 W; Figure S2: XRD patterns of lunar regolith simulant at different heat treatment temperatures.
