# Supplementary material for: Microwave-Sintered Lunar Regolith Bricks for Lunar Infrastructure: Fracture Behavior, Tribological Performance, and Electromagnetic Wave Transmission
Source: Materials (Basel). 2026 May 6;19(9):1907. doi: 10.3390/ma19091907 (PMC13164667; doi:10.3390/ma19091907)
Supplement: Supplementary file 1 [file materials-19-01907-s001.zip › materials-4240426-supplementary.pdf]

materials

MDPI

Supplementary Material

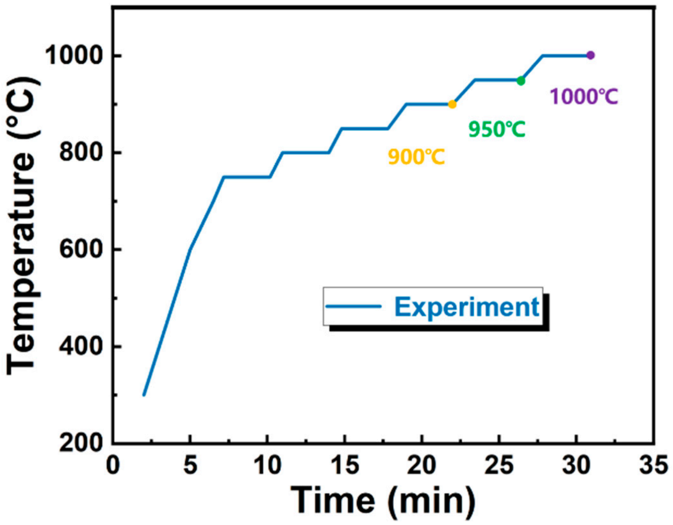

Figure S1. Temperature comparison between experiment at 3000 W.

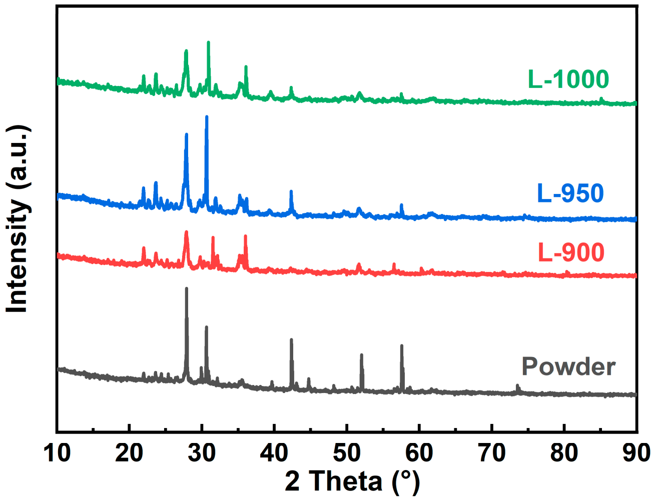

Figure S2. XRD patterns of lunar regolith simulant at different heat treatment temperatures.
